# A Prototype Exercise–Empowerment Mobile Video Game for Children With Cancer, and Its Usability Assessment: Developing Digital Empowerment Interventions for Pediatric Diseases

**DOI:** 10.3389/fped.2018.00069

**Published:** 2018-04-09

**Authors:** Carol S. Bruggers, Sabrina Baranowski, Mathew Beseris, Rachel Leonard, Derek Long, Elizabeth Schulte, Ashton Shorter, Rowan Stigner, Clinton C. Mason, Alisa Bedrov, Ian Pascual, Grzegorz Bulaj

**Affiliations:** ^1^Division of Hematology-Oncology, Department of Pediatrics, University of Utah, Salt Lake City, UT, United States; ^2^Primary Children’s Hospital, Salt Lake City, UT, United States; ^3^Spy Hop Youth Media, Salt Lake City, UT, United States; ^4^Department of Medicinal Chemistry, College of Pharmacy, University of Utah, Salt Lake City, UT, United States; ^5^Juan Diego Catholic High School, Draper, UT, United States

**Keywords:** exercise, video game, pediatric cancer, empowerment, pediatric chronic disease, digital health, mobile health, electronic health

## Abstract

**Background:**

Medical advances continue to improve morbidity and mortality of serious pediatric diseases, including cancer, driving research addressing diminished physical and psychological quality of life in children with these chronic conditions. Empowerment enhances resilience and positively influences health, disease, and therapy understanding. We describe the development and usability assessment of a prototype *Empower Stars!* mobile video game grounded in behavioral and exercise theories with the purpose of coupling physical exercise with empowerment over disease in children with cancer.

**Methods:**

Academic faculty, health-care providers, and community video game developers collaborated in this project. The iPadAir was selected as a delivery platform for its accelerometer and gyroscope features facilitating exercise design. *Unity* multiplatform technology provided animation and audiovisual features for immediate player feedback. Javascript, C#, Photoshop, Flash, and SketchUp were used for coding, creating graphical assets, Sprite sheets, and printing files, respectively. 3D-printed handles and case backing were used to adapt the iPad for physical exercise. Game usability, engagement, and enjoyment were assessed *via* a multilevel study of children undergoing cancer chemotherapy, their parents, and pediatric cancer health-care providers. Feedback crucial for ongoing game development was analyzed.

**Results:**

A prototype *Empower Stars!* mobile video game was developed for children 7–14 years old with cancer. Active, sedentary, educational, and empowerment-centered elements intermix for 20 min of exercise within a 30 min “one-day treatment” gameplay session involving superheroes, space exploration, metaphorical cancer challenges, life restoration on a barren planet, and innumerable star rewards. No player “dies.” Usability assessment data analyses showed widespread enthusiasm for integrating exercise with empowerment over cancer and the game itself. Favorite elements included collecting star rewards and planet terraforming. Traveling in space and the Healthy Food Choice game were least liked. The need for improved gameplay instructions was expressed by all groups. The usability study provided essential feedback for converting the prototype into alpha version of *Empower Stars!*

**Conclusion:**

Adapting exercise empowerment-promoting video game technology to mobile platforms facilitates usability and widespread dissemination for children with cancer. We discuss broader therapeutic applicability in diverse chronic pediatric diseases, including obesity, asthma, cystic fibrosis, diabetes, and juvenile idiopathic arthritis.

## Introduction

Recent advances in pediatric medicine have resulted in markedly improved patient survival, thus increasing prevalence of those living with diverse serious and previously often fatal pediatric chronic diseases, including cardiac, pulmonary, renal, autoimmune disorders, and cancer (1–5). Related physical and psychological comorbidities contribute to challenges of optimizing health and quality of life (QOL) for this growing population (2, 6–10).

Consider pediatric cancer as an illustrative example. Dramatic improvement in 5-year survival from 28% in 1960 to currently over 75% drives research to improve physical and psychological QOL from diagnosis through survivorship (4, 10). While cancer-directed therapies have been extensively studied, related *physical* QOL (strength, flexibility, cardiopulmonary function, endurance, and body habitus) and *psychological* QOL (mood, self-esteem, sense of control and vitality, body image, and family and peer relationships) dramatically affected during and after cancer treatment, have received comparatively limited attention (10–13). Exercise interventions are feasible yet limited in this population (14–18), and psychologic comorbidities are mainly addressed by counseling (12, 19, 20). A complimentary approach to addressing cancer-related physical and psychological comorbidities involves personal empowerment over disease (21). Empowerment is a core patient-centered, multidimensional process that potentiates one’s positive influence over personal heath by promoting resilience and restoring hope (22–24).

Video game technology and digital health [electronic health (eHealth) and mobile health (mHealth)] are emerging as effective empowerment-promoting tools by improving one’s understanding of health, disease, and treatment options (6, 21, 25–28). Video games show potential for health-related behavioral change by promoting disease education and self-management. Children with cancer who played Re-Mission™, a sedentary video game designed to improve disease education and therapy compliance, showed improved mood, cancer-related knowledge, and chemotherapy adherence (29, 30). Functional magnetic resonance imaging documented increased mesolimbic reward system activation while playing Re-Mission™ (31). Disease focused activity-promoting video games are primarily limited to adapting commercially available console systems and exer-games to target a specific population, like obesity and stroke (32, 33). To the authors’ best knowledge, no activity-promoting video games developed for children with cancer, or specifically for other pediatric diseases, currently exist. However, the few video games and apps designed expressly for pediatric oncology patients are limited primarily to a late-effects management app for cancer survivors (34, 35) and Re-Mission™ (30).

With these challenges and concepts in mind, we have been developing a video game technology that couples physical exercise with mental empowerment directed at simultaneous improvement of physical strength and endurance, and psychologic QOL of pediatric oncology patients (21, 36–40). To this purpose, a unique multidisciplinary team comprised of university faculty and an innovative local youth media program created a cancer-specific activity-promoting mobile video game prototype called *Empower Stars!* (38) The purpose of this paper is to describe: (1) the creation and unique aspects of the prototype *Empower Stars!* video game and (2) the results of the multilevel usability assessment conducted to provide essential direction for creating the alpha version of *Empower Stars!*

## Materials and Methods

### Design and Development of a Prototype *Empower Stars!*

Before developing the prototype *Empower Stars!* mobile video game, significant and essential groundwork had been laid in the process of creating an exercise–empowerment coupled video game using a PlayStation format (21, 36, 37, 40). The initial of concept of coupling physical exercise and mental empowerment as a video game intervention for children with cancer stemmed from several pivotal clinical observations: (1) the incredible determination and resilience of a child and their family members, when a child is diagnosed with and undergoes treatment for cancer, (2) the significant physical and emotional deconditioning that necessarily accompanies the months of pediatric cancer treatment involving surgery, radiation and chemotherapy, and its attendant adverse effects, and (3) the sense of empowerment exhibited by a child, whether young or older, when he or she overcomes a disease or treatment-related obstacle. Examples of overcoming cancer-related obstacles include bravery with portacath access, accomplishing magnetic resonance imaging studies without needing sedation, and taking those first steps following tumor resection and knee reconstruction. Additional motivation was derived from the increasing prevalence of significant adverse late effects, including decreased physical activity and increased incidence of obesity and bone demineralization that can occur following cancer treatment pediatric cancer survivors.

Discussions involving children with cancer and their parents, nurses, child life specialists, psychologists, physical therapists, social workers, pediatric oncology physicians, and video game developers confirmed interests to develop of an exercise–empowerment video game for children with cancer. Given the overwhelming support, the first iteration of an exercise–empowerment video game, The Patient Empowerment Exercise Video Game (The PE Game) was created (36, 37, 40). Creating The PE Game entailed regular and frequent face to face meetings with video game developers’ health-care providers and entrepreneurial faculty. A small IRB-approved usability assessment was conducted with patients whose hospitalization was anticipated to last longer than 21 days. Children, parents, and health-care workers enjoyed playing the game, and its underlying philosophy (unpublished data). However, the large cart apparatus, the complex computer-PlayStation interface, and the lengthy decontamination process between users were significant barriers that precluded further development.

Thus, our efforts were shifted toward developing a disease-specific video game promoting physical activity, empowerment, and behavioral change in pediatric cancer patients using a mobile tablet platform. This would facilitate broader technology dissemination in a relatively easy and affordable manner to children with cancer, and potentially other chronic pediatric diseases. Based on the aforementioned experiences, our efforts shifted toward developing a video game promoting physical activity, empowerment, and behavioral change in pediatric cancer patients using a mobile tablet platform. In collaboration with a local youth media organization, the prototype *Empower Stars!* video game was designed and created. The driving hypothesis in the development of this video game was: the integration of physical exercise with empowerment-promoting strategies can be accomplished on a *mobile platform* to create a disease-specific video game therapy for children with cancer. The creation of this prototype video game also involved a lengthy, multidisciplinary process involving of youth video game developers at Spy Hop Youth Media, academic faculty, and a team of health-care providers who specialized in the care of children with cancer—nurses, child life specialists, psychologists, social workers, physical therapists, and pediatric oncology physicians. Grounded in exercise, behavioral health, and video game engagement theories (27, 29, 41–46), the prototype *Empower Stars!* video game was designed to be interactive, engaging, exercise promoting, and empowering while also being easily portable and playable in diverse inpatient and outpatient settings (38). The iPad tablet platform was chosen for its portability, ease of use, wide availability, and its programmable accelerometer and gyroscopic motion control features that facilitate exercise element design and performance in standing, sitting, and semi-supine positions. This game development process entailed regular group meetings to discuss ideas, progress, pitfalls, and solutions. Children and their parents were also periodically queried informally for their input regarding storylines and characters during the early phases of the game development. Proposed changes were then incorporated into the video game prototype and subsequent iterations were evaluated by health-care providers and academic faculty.

The game was designed using Unity Multiplayer game engine (47), a powerful, flexible development platform employing a 64 bit editor for creating multiplatform 2D and 3D games and interactive experiences across diverse stationary and mobile platforms. This platform also served as a testing environment and provided immediate feedback during developer design and testing before targeting export to the iPad. Integrative dynamic graphic and sound mixers, and animation features linked with audiovisual effects provide immediate real-time speed, direction, and acceleration player feedback. Coding was done in Javascript and C# (48). Graphic assets and Sprite sheets were made in Photoshop and Flash (49), respectively. Files for 3D printing were created using SketchUp Free software (50). Graphics, animation, and components were set to trigger events and interactions between users and the game. Music and Audio files were generated using Ableton Live (51), ProTools (52), and Garage Band (53).

The multidisciplinary team collaborated to create an imaginative story line that included metaphorical mini-game exercise challenges, educational and psychosocially empowering experiences. Audiovisual and kinetic feedback and rewards were designed to enhance enjoyment and immersion. Design elements deemed crucial to facilitate game flow during gameplay were: imaginative stories and graphics embodying empowerment over cancer; avoidance of violence and negative, death-related themes; clear instructions; and inclusion of fun, engaging, and non-repetitive exercise elements of ample intensity to raise heart rate. The unique power bar handles and backing were designed and produced using a 3D printer and then affixed to a commercially available iPad case to accommodate diverse physical exercises while simultaneously providing iPad device protection.

### Empower Stars Usability Assessment

**Figure 1:**
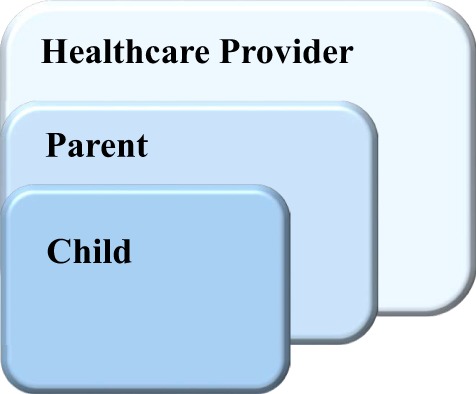
The prototype *Empower Stars!* video game usability assessment centered on a multilevel user approach that included children with cancer ages 7–14 years of age, one of their parents, and Pediatric Hematology-Oncology health-care providers.

**Figure 2:**
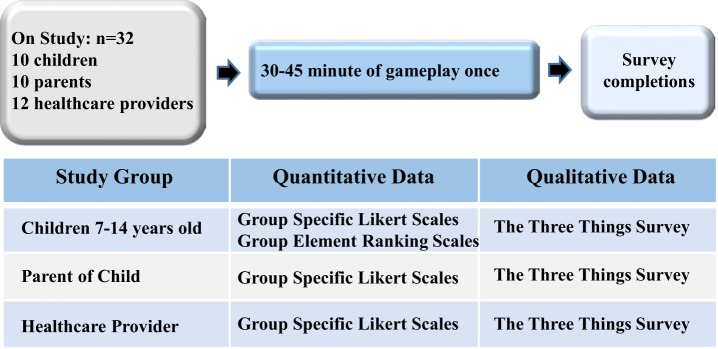
The prototype *Empower Stars!* video game usability assessment design involved a single gameplay session by three distinct yet related study populations as previously detailed, and included collection and analysis of both quantitative and qualitative data.

The driving hypotheses for assessing the prototype *Empower Stars!* video game usability were (A) the video game would be engaging and usable and (B) important feedback collected in this usability assessment will provide positive and negative feedback critical in the creation of the alpha video game. Both qualitative and quantitative data were collected in this University of Utah Institution Review Board-approved study to obtain constructive feedback in the areas of usability, engagement, and enjoyment that were deemed critical for future game development and improvement (43, 54, 55). With an underlying premise that successful and optimal use of a new therapeutic intervention would require endorsement by potential users and prescribers, a multilevel approach that could yield essential feedback from each of these groups was applied to the usability assessment design. Thus the multilevel subject population consisted of three groups: (1) 10 children ages 7–14 years of age who were undergoing chemotherapy to treat their respective cancers, (2) one of the parents of each child, and (3) 12 pediatric health-care providers, including nurses, child life specialists, social workers, and physicians, all from Primary Children’s Hospital and University Pediatric Hematology-Oncology Division (Figure 1). For each study participant, the Research Assistant introduced the prototype *Empower Stars!* game technology and game sequences on the iPadAir. Participants then played the prototype *Empower Stars!* video game for a single 30–45 min gameplay session. Immediately after completing gameplay, each participant filled out the appropriate study group qualitative and quantitative evaluation tools. Evaluation tools for the children included: a 15 statement survey tool with statements appropriate for children centered on usability, engagement, and enjoyment using a 5-point Likert scale with response anchors; a picture survey ranking most to least favorite for eight game elements and five mini-games; and a simple open-ended survey tool called “The Three Things.” Evaluation tools for the child’s parent who either played or observed their child playing the prototype *Empower Stars!* video game, included a 10 statement survey tool with statements appropriate for parents, using a 5-point Likert scale with response anchors and “The Three Things” open-ended survey tool. Evaluation tools for health-care providers included a 10 statement survey tool with statements appropriate for health-care providers, using a 5-point Likert scale with response anchors, and “The Three Things” open-ended survey tool (Figure 2).

### Statistical Analysis

Five-item Likert scales with end anchors were used to assess child, parent, and provider opinions regarding usability, enjoyment, and engagement. Ratings from the Likert scale were recorded and transformed to a numerical scale of 1–5. Tests for significant difference in ratings were assessed by a two-sided Wilcoxon–Mann–Whitney test. SAS (SAS Institute, Cary, NC, USA) was used for statistical comparisons. GraphPad Prism (GraphPad Software, La Jolla, CA, USA) was used to generate the accompanying figures.

The children’s assessment also contained two visual ranking scales for ranking of likability of (1) the eight main game environments and characters and (2) the five exercise mini-games and games. Mean scores were determined for each scale.

Each study participant was also asked to complete a simple open-ended questionnaire entitled “The Three Things” to provide feedback regarding the three things they liked the best, the three things they liked the least, and the three things they would change about the video game. Answers that appeared at least two times were compiled and presented in a summary table.

## Results

### Video Game Creation

**Figure 3:**
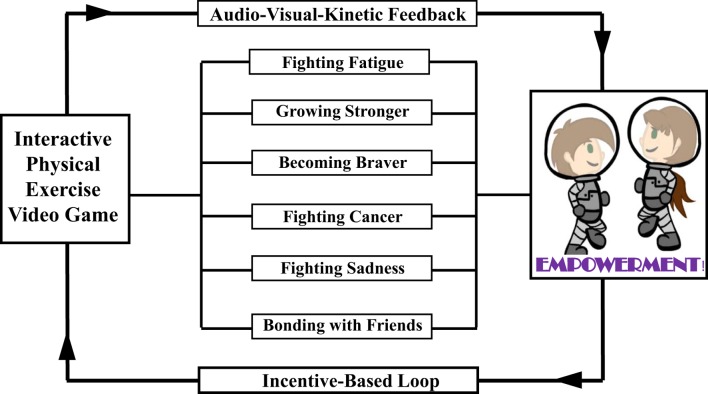
The prototype *Empower Stars!* video game is grounded in behavioral, empowerment, exercise, and engagement theories. It serves as a promising prototype intervention that couples exercise with empowerment over a specific disease to positively influencing health, disease, and therapy understanding in children with cancer and other chronic pediatric diseases.

The prototype *Empower Stars!* video game was created for children ages 7–14 years with cancer undergoing chemotherapy. The initial focus was to create a proof-of-concept prototype to deliver a “one-day” intervention that consisted of 30 min of gameplay time which included 20 min of physical exercise using iPadAir and a specialized hardware case. Coupling physical exercise and mental empowerment as a video game intervention for children with cancer and other chronic diseases has been previously defined (21, 36, 37, 39, 40). This innovative approach is based on creating an audio–visual-kinetic feedback loop bridging physical movements with empowering messages and incentives (Figure 3).

#### Story Line

**Figure 4:**
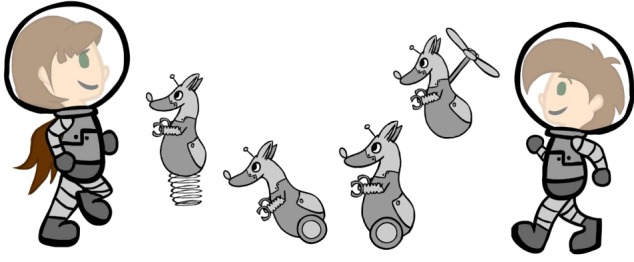
Together, the Space Explorer Hero avatar and the companion super buddy Keemo overcome many obstacles on their space mission to restore life to the barren planet and conquer the Cercer crabs before safely returning to the space station. The Space Explorer Hero is customizable for gender, and Keemo has multiple mobility options, all of which are used during gameplay to overcome different obstacles during the mission.

There are five environmental settings in this imaginative space exploration story. The game begins at the “Space Station,” where the Space Explorer Hero avatar, customizable for gender, prepares for the journey by completing two different exercise mini-game challenges and one nutrition education exercise mini-game challenge. The Space Explorer Hero then boards the spaceship and begins “the Space Flight” that involves an exercise mini-game where the space ship must be maneuvered through rings of stars, where additional star reward are earned. The space ship then stops at “the Outer Planet Puzzle” where the Super Hero meets up with Keemo, the Super Buddy avatar with multiple mobility options, each of which is needed to overcome different game obstacles at various time points during gameplay (Figure 4). Together, these two heroes work together to solve the Outer Planet Puzzle by overcoming diverse obstacles. The energy needed to accomplish this task is earned by successfully completing several 30 s exercise periods at “power-up stations.” These power-up stations are strategically placed throughout the Outer Planet Puzzle. Once the puzzle breaks apart into hundreds of tiny fragments, the Super Heroes are able to enter “the Positron Planet Core” where the positrons are gathered together in another exercise mini-game challenge. The Space Explorer Hero and Keemo are then able to land on the planet surface and begin “Terraforming the Planet” by bringing clouds, water, trees, and new life forms to the surface of the planet, and overcoming the Cercer crabs. Innumerable star rewards are collected during terraforming process. Once the planet terraforming is complete, and the Cercer crabs are all destroyed, the Space Explorer Hero once again boards the space ship for travel back to the Space Station and again navigates through the star rings before successfully landing back at the space station.

**Figure 5:**
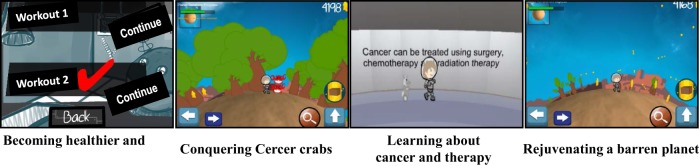
Physical, educational, and emotional empowerment themes are prominent throughout the prototype *Empower Stars!* video game. These themes include exercising to become stronger; conquering Cercer crabs that are metaphoric for cancer; learning about cancer, treatment and supportive care *via* educational messages and educational mini-games; and restoring life a barren planet by creating clouds, running water, plentiful trees, and new life form, a process summarized as “terraforming.”

Game activity-associated empowerment themes are integrated throughout the game, and center on overcoming diverse obstacles metaphoric for pediatric cancer challenges (Figure 5). Extrinsic and intrinsic glory rewards include innumerable collectible bright stars, achievement-related auditory sounds to reinforce feelings of accomplishment, numerical account of stars earned, numerical and bar graph visual indicators of progress toward terraforming the planet, conquering the Cercer crabs, and achieving empowerment over the metaphoric cancer challenges. While the initial prototype *Empower Stars!* video game has one planet with diverse challenges and exercise components yielding a “one-day treatment,” the number of planets in this universe is infinitely expandable, and each planet can have unique exercise mini-games and audio–visual effects for increased game level number and complexities, challenges, and rewards.

#### Physical Exercise

**Figure 6:**
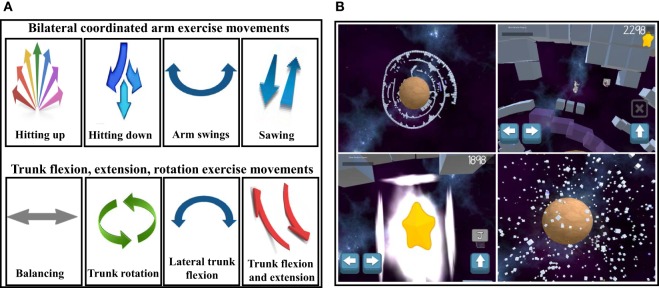
The programmable iPadAir accelerometer and gyroscopic motion features facilitate incorporation of specific upper extremity and trunk exercise motions for **(A)** exercise motions needed to overcome a specific mini-game challenge and **(B)** exercises for 30 s “power-ups” to provide the energy needed to continue gameplay, as illustrated in the Planet Puzzle mini-game sequences.

Physical exercise movements are designed to increase upper extremity and trunk strength, flexibility, and mobility, while being fun, natural, and comfortable (Figures 6A,B). Two distinct exercise categories are utilized. The first category of exercises involves movements that are an end into themselves, as in iPad manipulation used to separate healthy from unhealthy foods nutrition mini-game, and iPad manipulation needed to maneuver the space ship through star circles during space travel. The second category of exercises involves exercise movements that last for 30 s at “power-up” exercise stations that are strategically located throughout the game. During these “power-ups,” innumerable star rewards are collected, and the player is rewarded with the energy needed for continued gameplay. A total of 20 min of exercise is distributed within the 30 min game play session. The 30 s time period for exercise was chosen based on players feedback provided during the game development. This was ample time for a player to feel some initial muscle fatigue without stopping the exercise altogether. The intensity of performing the exercises varied with the specific player. Those who were feeling relatively well in general, usually a subject in the outpatient setting, exercised quite vigorously. In contrast, a patient who was hospitalized generally did not feel as well, and thus often did not perform the exercises with as much vigor. The faster a player moved during the exercise games, the more star rewards were collected. However, the game was designed such that only a limited iPad tablet motion excursion was necessary to trigger star reward collection.

#### Educational Empowerment Content

**Figure 7:**
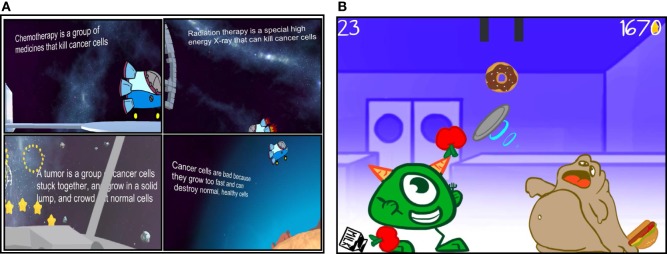
Educational empowerment is facilitated by **(A)** important on-screen health-related messages interspersed through the game and **(B)** mini-games that center on teaching important components of cancer patient care, like nutrition, that are preceded by simple graphic gameplay instructions.

Educational empowering content addresses such questions like: “What is cancer?” “What is chemotherapy?” “What is radiation therapy?” “What are treatment side effects?” “How can I improve my nutrition during my cancer treatment?” and “How can I help myself get stronger and healthier?” This content was presented in two different formats in the prototype *Empower Stars!* video game. The first educational format involves written messages that address several important cancer-related and supportive care-related facts. These messages are appear on the iPad screen and then move across the screen at distinct time points during gameplay. The second educational format incorporates specific content into an actual exercise mini-game challenge, like the Food Nutrition mini-game (56, 57). The Healthy Food Choice exercise game included three “healthy food choices”—apples, carrots and milk, and three “unhealthy food choices”—deep fried sugar glazed donuts, soda pop, and grilled hamburgers, as defined by The American Heart Association (58). To play this game, the player needs to move the iPad in different directions to successfully separate healthy foods from unhealthy foods as the food items drop down from the top of the screen (Figures 7A,B).

#### Design and Fabrication of iPadAir Hardware Case

**Figure 8:**
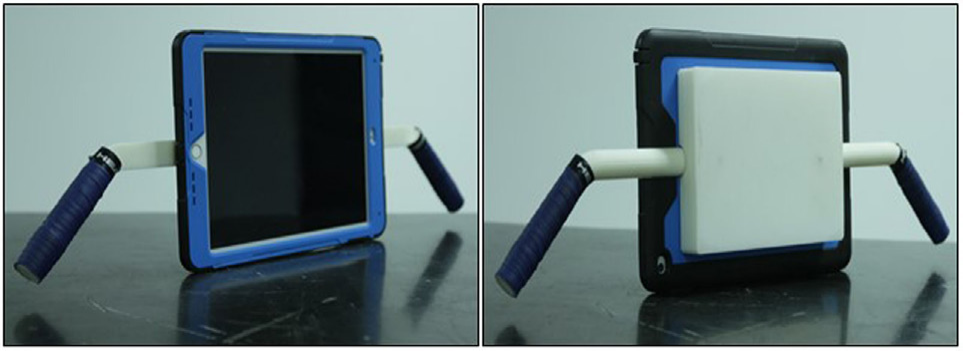
The light weight, high density bi-handled casing support pieces were created using a 3D printer and then affixed to a commercially available iPad case. This adapted casing facilitates active exercise while simultaneously protecting the iPadAir.

Two light weight, high density ergonomically designed side handles and a rectangular shaped backing plate were printed at the University of Utah 3D printing facility. These were then securely affixed to a commercially available iPadAir case to promote motion/sensor accuracy in addition to providing iPad protection during physical exercise (Figure 8).

### Assessment of Game Usability, Engagement, and Enjoyment

#### Children

**Figure 9:**
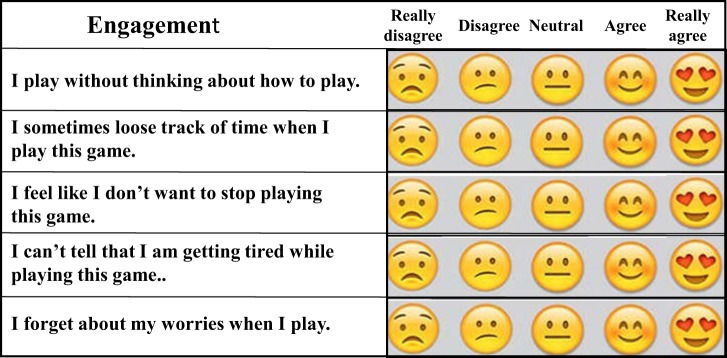
For pediatric subjects, three 5-point Likert scale assays, each consisting of five questions, were used to asses usability, engagement, and enjoyment of the prototype *Empower Stars!* video game. Emoji faces were used instead of five numbers to indicate the degree of subject agreement or disagreement with the statement made, as illustrated here in the section evaluating engagement.

**Figure 10:**
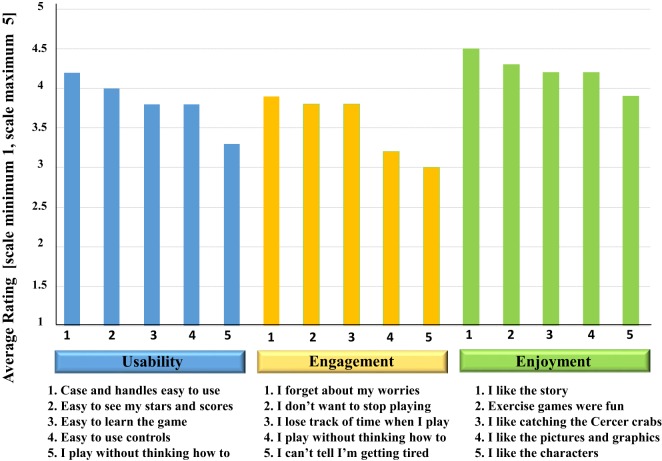
Mean responses of 10 pediatric cancer children to questions related to usability, engagement, and enjoyment of the prototype *Empower Stars!* video game. Ratings from the Likert scale from each of the three areas assessed were transformed to a numerical scale of 1–5. The mean rating scores for each question in the categories of usability, engagement, and enjoyment were then plotted.

The questions asked to the children pertained to their relationship to cancer-related effects and to their experience playing the game with respect to usability, engagement, and enjoyment (Figure 9). These questions were unique from those posed to the parents and the health-care providers. The overall highest average rating by the pediatric cancer children related to their liking of the story in the games. The two lowest average ratings by the children were their responses to questions of their not noticing their getting tired while playing the game and to the question related to their being able to play without thinking about how to play the game. These responses may characterize the general temporary enjoyment the children had while being engaged in this activity, yet some disease-related effects could not be entirely overcome by its being played (Figure 10).

**Figure 11:**
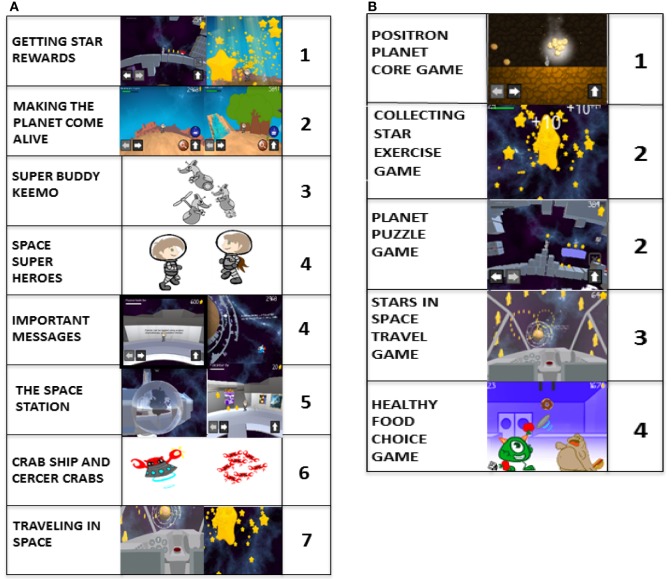
Mean responses of the 9 of 10 pediatric subjects who were able to rank-order two distinct picture scales. **(A)** Of the eight major game components, collecting the star rewards was the most liked (1) and traveling in space was the least liked (7). The Space Heroes and the important messages were tied for the fourth most liked. **(B)** Of the five distinct exercise mini-games, the Positron game was the most liked (1) and the healthy food choice game was the lease liked. The star exercise mini-game and the planet puzzle game tied for the second most liked.

Results of the Picture Tool assay showed that the two favorite game components were collecting star rewards and terraforming the planet. Traveling in Space from the space station to the planet was the least favorite game component. The two most liked exercise mini-games were the Positron Planet Core Game and the Collecting Stars Exercise Game, while the least liked exercise mini-game was the Healthy Food Choice exercise game (Figures 11A,B).

#### Parents and Health-Care Providers

**Figure 12:**
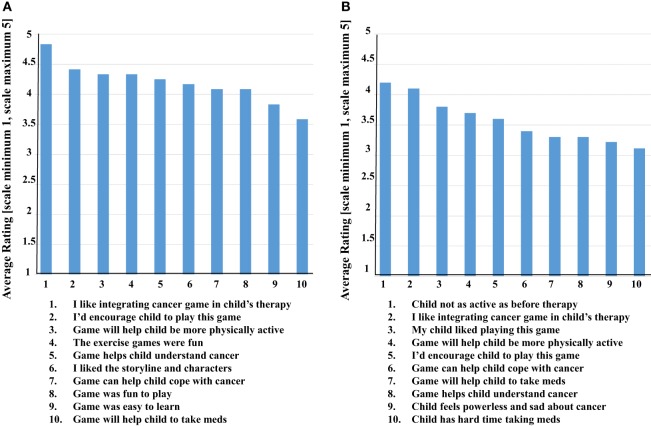
Health-care providers and parents each completed a 10 question Likert scale, which contained five unique and five overlapping questions. **(A)** The mean ratings of health-care providers (*n* = 12) were above 3.5 in all categories, with the top-rated being liking the idea of integrating a cancer-focused video game into the overall therapy for a child with cancer. **(B)** The mean ratings of parents (*n* = 10) were somewhat lower though all still above 3.0. The top-rated statement identified their child is not being as physically active as before the diagnosis and initiation of cancer treatment, followed very closely by liking the idea of integrating a cancer-focused video game into the overall therapy for their child with cancer.

**Figure 13:**
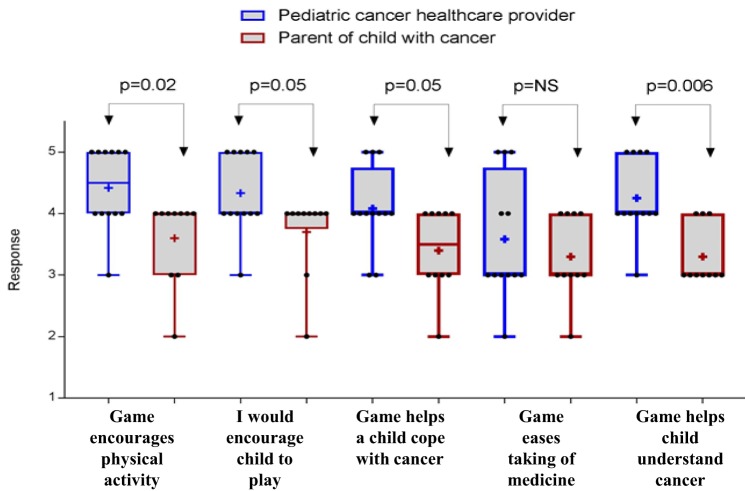
Five questions were asked to both health-care providers and parents of the children with cancer. Ratings were transformed to a scale of 1–5, and significant differences were assessed with a two-sided Wilcoxon–Mann–Whitney test. The “+” signs in the box-and-whiskers plots indicate the mean response of each group, box extends from the 25th to 75th percentiles, and a vertical line (when visible) drawn at the median response level.

The prototype *Empower Stars!* video game was positively received by health-care providers and parents, with all mean scores for all statements being above 3.5 and 3.0, respectively (Figures 12A,B). Five of the questions given to health-care providers for the pediatric cancer patients were identical to those given to the parents of the children with cancer. While no significant difference existed between parents and providers with regard to the question of whether the game would make the taking of medicines easier for the children, the rest of the questions was borderline significant or completely significantly different (Figure 13). For all such questions, the mean and median responses were lower by the parents. The most striking significant difference was in response to the question of whether the game would help children understand cancer better, with a mean rating difference of 1 level higher in providers.

#### “The Three Things” Open-Ended Survey Tool

Responses from children, parents, and health-care providers to this open-ended questionnaire yielded very useful information. In general, the prototype *Empower Stars!* video game was well received by each group. Children liked the stars, exercises, storyline and characters, and cancer facts, although the instructions provided within the game for knowing what to do and how to do it were not adequate. They also wanted more characters, character actions, and challenge levels. Parents and health-care providers liked incorporating the exercises and integrating the exercises with empowerment themes, both educational and psychological. All groups found instructions regarding what to do and how to do it lacking, and the overall objective of the game to be unclear (Table 1).

**Table 1:** Ten children, 10 parents, and 12 health-care providers completed “The 3 things…” simple sentence completion tool after playing or observing their child play *Empower Stars*.

	The 3 things I liked most	The 3 things I did not like	The 3 things I would change
Children	The Star Rewards The exercises The characters The cancer facts The story It was fun It was something new	Characters were too cartoony Exercises were too hard It was hard to know what to do sometimes Instructions were not good	Add more detailed instruction Add more levels Add more cancer crabs Add more actions for the heroes Add more actions for the crabs Add more characters Add more music choices
Parents	Physical exercise and game interactions Idea of teaching about cancer Mind stimulation and education The handles The exercises Helps kids see cancer can be destroyed	Hard to understand at times Instructions not very clear Had to be able to read to play	Improve the instructions Make attack on cancer more direct Emphasize the exercises more Make goals to be accomplished clearer More celebration
Health-care providers	The idea of empowerment over cancer Incorporating the education elements How interactive the game is Portability You have to move to play The storyline and characters	Hard to understand at times Some levels were long and repetitive Music was the same throughout Game objectives sometimes not clear Not enough levels Did not understand the terraforming tools	Clarify and expand the instructions Add more education, including radiation and surgery Emphasize the exercises more Add more animation to crabs and superheroes Make the awards more distinctive Add more ethnicity choices to characters

## Discussion

Video games provide a fun, appealing format that captivates a player’s mind and body, regardless of age. The concept of empowering children to fight their cancer using an activity-promoting video game that directly couples physical exercise with personal empowerment over cancer *via* visual, kinetic, and auditory feedback is a novel approach (21, 36–40). Digital technologies are currently focused on delivering either physical exercise or psychological interventions, whereas a direct coupling of these two modalities to create and reinforce a reward feedback loop in the patient’s brain is innovative. Simultaneous engagement of neuromuscular, neuroendocrine, and cardiovascular systems *via* exercise–empowerment videogames warrants translational research.

This concept was first explored in The PE Game. The PE Game prototype was built on a PC communicating with Sony PS3 running Sony’s Move.me software and was designed to be played within a small hospital inpatient room. It consisted of a series of discrete metaphorical mini-games centered on overcoming cancer-related challenges, and integrated physical exercise with promoting teamwork, a fighting spirit, and a sense of personal empowerment in children with cancer (36, 37, 39, 40). While enthusiastically embraced by patients, parents, and health-care workers, the complexities of the computer and PlayStation interfaces were complex and not user-friendly, and the large PE Game cart apparatus was challenging to transport and disinfect (Bruggers and Bulaj, unpublished data). This prevented independent and spontaneous game plays at a time when a patient might feel most physically able and psychologically motivated to play. This same patient group may well constitute those who may be most likely to benefit most from an exercise–empowerment intervention. Thus based on this same exercise–empowerment concept, our research and development efforts focused on using a mobile platform which has the potential for easy and independent patient usability and more widespread dissemination to patients with cancer and other chronic pediatric diseases.

### Multilevel Usability Assessment

The multilevel usability assessment of the prototype *Empower Stars!* video game served the planned purpose of providing important feedback information for game improvement. The prototype *Empower Stars!* video game was enthusiastically received by children with cancer, their parents, and their health-care providers. Such common endorsement by all planned participant groups is essential when introducing a new adjuvant therapy, including the children who will be regularly playing the game, their parents who provide ongoing daily care and encouragement, and their cancer treatment providers who are responsible for design, implementation, and continuous cancer treatment program monitoring. It is clear, however, that significant improvement in game usability with respect to clarifying different aspects of the game and gameplay instructions will need significant improvement in the next version of the game.

Health-care providers were particularly keen on the idea of incorporating cancer education and empowerment and exercise in a portable disease-specific video game. Parents also liked the education, the exercises, and that the game actually helped show their children that cancer can be destroyed, with some wanting an even more direct and less metaphorical attack on cancer. The most striking significant difference between parents and health-care providers was in response to the question of whether the game would help children understand cancer better, with a mean rating difference of 1 level higher in providers. This may reflect the providers’ comprehension of cancer facts being provided in the game which was not as well taken in by the parents. In general, the overall lower rating of parents may also potentially reflect an understandably more pessimistic reflection on rating anything. Ideally, future surveys would include a few sample questions not related to cancer which could assess such a general difference in this group.

### Pediatric Cancer Patients, Video Games, and Empowerment

Playing commercially available video games can decrease chemotherapy-associated nausea in oncology patients when used as distraction therapy (59), and playing virtual reality computer games as add-on therapy improves depressive symptoms. Recently developed mobile apps address empowerment and managing late effects associated with successful childhood cancer treatment (34, 35). Playing the sedentary videogame Re-Mission™ resulted in significant improvements in both disease understanding and medication compliance in a large randomized, prospective clinical trial of teens and young adults with cancer (29–31), and it has been well documented that medication adherence is positively correlated with cancer cure in children with acute lymphoblastic leukemia (60). Although limited in number, such apps and video games provide important evidence that carefully designed video games can enhance specific behavioral outcomes like increasing knowledge and compliance, and potentially promote information seeking in children with cancer (29). The potential contribution of *mobile* disease-related interventions is illustrated by the development of Re-Mission 2™ (61), a mobile online sedentary video game for teenagers with cancer that promotes empowerment, self-efficacy, and positive emotions, while also encouraging medication adherence. By contrast, “That Dragon, Cancer” is a recently created video game that powerfully reveals the challenges, hopes and realities involved in the world of a child and his family after being diagnosed with cancer (62).

### Pediatric Chronic Disease as Targets for Exercise–Empowerment Interventions

An estimated twenty percent of children are affected by some type of chronic disease. In contrast to the treatment of acute self-limited illness, children with chronic diseases have a more prominent role in directing their own care, with parental guidance, and in making ongoing changes in how they live their lives, particularly as they transition into adulthood (63). As summarized in Table 2, the broader applicability of a disease-specific exercise–empowerment pediatric video game becomes evident when one studies the shared challenges and potential intervention targets of representative chronic pediatric diseases. The development of the exercise–empowerment digital intervention to diverse chronic pediatric disease is beyond the scope of this paper. However, the potential applicability for such interventions becomes apparent when reviewing selected pediatric diseases as illustrative examples.

**Table 2:** Diverse pediatric chronic diseases share similar psychosocial targets for empowerment intervention, and potential exercise benefits that could be addressed with disease-specific exercise–empowerment interventions like the prototype *Empower Stars!* video game.

Chronic pediatric illness (reference)	Psychosocial targets for empowerment intervention	Benefits and safety of physical exercise
Obesity (33, 64–68)	Exercise and diet non-compliance Social isolation Low self-esteem Limited self-management	Improved HR-QOL Improved cardiopulmonary status Improved self-efficacy Decreased comorbid chronic diseases
Asthma (69–74)	Medication non-adherence Limited disease understanding Limited self-management	Improved quality of life (QOL) Improved therapy compliance Reduced disease symptoms Safety concern: asthma exacerbation
Cystic fibrosis (1, 75–80)	Therapy program non-adherence Limited disease understanding Limited self-management	Improved pulmonary function and fitness Improved HR-QOL Improved therapy compliance Safety concern: shortness of breath
Chronic renal disease (9, 81–89)	Medication non-adherence Limited disease understanding Limited self-management Social isolation; low self esteem	Improved therapy compliance Improved bone health Improved growth/development Improved HR-QOL
Congenital heart disease (5, 90–93)	Limited disease understanding Limited self-management Feelings of fear, uncertainty Medication non-adherence	Improved cardiovascular status Improved muscle strength Improved HR-QOL Safety concern: heart strain with exercise
Diabetes (28, 94–99)	Medication non-adherence Limited disease understanding Limited self-management Self-esteem, depression	Improved glycemic control Improved HR-QOL Improved weight control Safety concern: induced hypoglycemia
Epilepsy (100–104)	Medication non-adherence Limited disease understanding Sense of hopelessness Depression, anxiety	Improved HR-QOL Improved depression Improved self-esteem Safety concern: exercise induced seizure
Juvenile idiopathic arthritis (25, 105–110)	Fear of uncontrolled pain Social isolation Low self-esteem Therapy program non-adherence	Increased range of motion Increased bone mineralization Improved HR-QOL Reduced joint inflammation and pain

Obesity is a complex multifactorial chronic disease of increasing prevalence that also greatly increases risk for other chronic diseases, including depression, type 2 diabetes, obstructive sleep apnea and hypertension While hereditary factors, ethnicity and particular socioeconomic and sociocultural conditions exacerbate the risk and severity of obesity, some modifiable personal behaviors, like physical exercise expenditure, sedentary and screen time, and dietary intake, also play a significant role in disease severity (64). Compared with playing sedentary videogames, playing active video games, like Dance Dance Revolution and Wii Sports, significantly increases energy expenditure, often to levels comparable to moderate-intensity treadmill walking (33, 66, 67). Moreover, participation in exercise video games by both overweight and healthy weight children have shown improved exercise self-efficacy at 2 years later (68). In children with asthma, adherence to treatment programs is challenging (69, 70). Web-based programs have shown efficacy in promoting asthma treatment adherence (71, 72). Although a potential trigger for asthma symptom exacerbation, exercise can improve disease symptoms and psychosocial adjustment (73, 74). Of note, control of asthma symptoms was improved after 8 weeks of exercise training delivered by an active video game (111). For children now diagnosed with the multisystem complex disease Cystic Fibrosis, the median predicted age of survival age is now into the sixth decade (75–77). However, young adults with Cystic Fibrosis self-report anxiety, depression, and poor adherence to recommended therapies, including exercise interventions (78), even though participation in exercise and education programs, improve aerobic capacity, nutritional status, and QOL (78–80). Chronic renal failure arising from diverse etiologies results in impaired growth, cardiovascular disease, and diminished physical and psychosocial QOL (9, 81–83). Exercise improves cardiovascular function and exercise capacity (84, 85) and psychosocial interventions, including eHealth, promote disease knowledge, compliance, and empowerment in these patients (86, 87). For children with congenital heart disease, participation in both hospital and home-based cardiac physical activity programs is safe and feasible and improves QOL and exercise capacity (90–92, 112). Finding proactive, engaging ways that promote physical activity and empowerment is important in promoting disease self-management as these teens transition into adulthood (5). Effective management of type 1 diabetes in children entails diligent monitoring of diet, medication, and physical activity (94, 95) and is complicated by diverse psychosocial factors including the desire for independence and social acceptance (96, 97). Exercise improves glycemic control and cardiovascular function (98), and educational interventions like video games and web-based programs improve diabetes knowledge, self-management, and QOL (28, 99). In children with epilepsy, disease-associated depression and anxiety occur more commonly than is characteristically seen in most other pediatric chronic illnesses (100). Psychosocial and exercise interventions improve self-knowledge, treatment compliance, seizure control, cardiovascular fitness and self-esteem in children with epilepsy (101–104). In Juvenile Idiopathic Arthritis, which is characterized by pain, fatigue, stiffness, and diminished exercise capacity, sustained adherence to exercise and medication programs is very challenging (105–108). Web-based self-management interventions show promise in improving treatment adherence, symptom control, and overall QOL (25, 109, 110). Common overlapping themes include: the feasibility and benefits of physical exercise and increasing disease-related knowledge; the shared existing psychosocial challenges; the challenges associated with transition from pediatric to adult health care; and the emerging, beneficial roles of eHealth and mHealth. Taken together, these data indicate a potential benefit to strategies aimed at improving exercise adherence, promoting personal empowerment, and development of disease self-management in children and teens as they enter adulthood (Table 2).

### Digital Empowerment Tools and eHealth and mHealth

Health-promoting interactive technologies show significant potential for effecting behavioral change and promoting disease self-management by education and active patient involvement directed at understanding health, disease, and treatment (6, 21, 42, 86, 87, 113–115). Several available electronic empowerment-promoting tools, including compact disk read-only memory, web/Internet-based interventions, and video game technology provide informative sources for education and empowerment with widespread appeal (6, 21, 29, 34, 116, 117). Serious sedentary video games focus on education and disease self-management of a specific health condition by merging lessons and quizzes with puzzles and plots, like *Packy and Marlon* which is designed to improve self-management in people with diabetes (118). Most health-related *activity-promoting* video games adapt commercially available console systems like Wii-Nintendo, Xbox-Microsoft, and PlayStation-Sony. Their respective commercially available “exer-games” are then played in a non-game context to target a specific population, like obesity (33). Wii™ Bowling, Wii™ Boxing, and WiiFit™ balance board improve balance, coordination, strength and general neurologic function in patients with Parkinson’s disease, stroke, and multiple sclerosis (32, 119–122). However, such interventions specifically designed for patients with a given disease to promote physical movement are still very uncommon.

Video games are also finding their place in the management of specific mood disturbances. Adults with depression randomized to treatment with add-on video game therapy showed significantly fewer depressive symptoms at 1 month compared with the control group (123). Playing prescribed casual video games reduces anxiety (124). Depressed adolescents treated with computerized cognitive behavior therapy using SPARX, an online computer program designed to help young persons with mild to moderate depression, showed significant improvement in depression rating, equal to those treated with traditional face to face cognitive counseling (125). “Zora,” a multiuser Internet-based psychosocial intervention designed for children undergoing hemodialysis and organ transplantation, provides a safe, beneficial virtual city/community support network for coping and sharing experiences (86).

Several challenges to successful development and widespread dissemination of video games for positive health-care behavioral currently exist (27). It is essential to assemble a multidisciplinary team that includes experienced video game designers as well as medical and behavioral health-care professionals with focus on the specific population and health-related concern. This is especially important in pediatric, given the chronological and developmental diversity within the pediatric population. Adapting a video game designed for healthy children can be problematic when played by children with a life-threatening disease if characters “die” when unable to overcome game challenges (126). Designing informative tools for objective outcome efficacy assessment is challenging, especially when efficacy assessment using placebo-controlled blinded clinical trial methodology is not feasible. Rapidly evolving video game technology is costly, and funding for development of serious video game targeting a specific health-care need, including pediatric cancer, is scarce.

It is encouraging that the U.S. Food and Drug Administration recently cleared digital therapeutic technologies, including BlueStar^®^ (WellDoc, Baltimore, MD, USA), a mobile platform promoting self-management in diabetes, the mobile medical app reSET™ (Pear Therapeutics, Boston, MA, USA) for the treatment of substance use disorder, and the Jintronix Rehabilitation System^®^ (Montreal, QC, Canada). Significant improvement in attention was seen in children with attention deficit hyperactivity disorder (ADHD), after 4 weeks of playing EVO (Akili Interactive, Boston, MA, USA), an iPad-based intervention designed for children with ADHD (127). Finally, in September 2017, the U.S. Food and Drug Administration announced a new digital health software pre-certification pilot program as part of a Digital Health Innovation Action Plan designed to promote digital health innovation while simultaneously promoting public health.

One attractive development pathway for the prototype *Empower Stars!* video game is to expand the current “one-day” prototype game version into the alpha version designed to deliver a multiday exercise–empowerment intervention for children with cancer. This would involve continued multidisciplinary collaboration among pediatric oncology care providers and video game developers to address the limitations of the current prototype. Results obtained from the 32 subject multilevel usability *Empower Stars!* assessment will be incorporated into the next version, with additional rounds of multilevel feedback obtained followed by a second multilevel re-assessment will be conducted to create the alpha version. Subsequent clinical testing of the alpha *Empower Stars!* would then allow identification of potential clinical benefits in children during and following chemotherapy, Incorporation of individualized goal setting, a powerful component of behavioral change, may also be feasible (128, 129). Once clinical efficacy can be demonstrated in the pediatric oncology population, then expansion of this mobile exercise–empowerment video game concept to other pediatric chronic diseases would be feasible. As illustrated in Table 2, there are several possible pediatric indications where “repurposing” the prototype *Empower Stars!* video game can lead toward adjunct digital therapeutic interventions in these and other pediatric chronic diseases. Development of video games as a digital therapeutic intervention is based on “software as medical device” pathway and is subject to regulatory compliance and pivotal clinical testing.

## Conclusion

Creating hope, improving QOL, reducing stress, and increasing one’s sense of control over the seemingly uncontrollable are all important components of patient empowerment over disease. The prototype *Empower Stars!* video game defines the novel approach of directly coupling physical exercise with promoting personal empowerment over cancer using a mobile platform. Learning that children like and will play such a game, that parents endorse the incorporation of such a game into their child’s treatment program, and that there is strong approval by health-care providers together all support continued development of a disease-specific exercise–empowerment video game as adjuvant medical treatment. Continued multidisciplinary collaboration with incorporation of serial multilevel user feedback assessment is essential for the development of the alpha version of *Empower Stars!* video game that can then undergo efficacy evaluation in children with cancer. Once this has been accomplished, the stage is set for developing similar exercise–empowerment mobile video game interventions personalized for diverse pediatric chronic diseases. Coupling physical exercise with personal empowerment in a judiciously designed and disease-specific video game that is created on a mobile platform provides the opportunity for widespread, affordable application to children with cancer, and serves as a prototype for management of diverse chronic diseases across many ages.

## Ethics Statement

The Usability Assessment study was reviewed and approved by the University of Utah Institutional Review Board and classified as a Minimal Risk Study. Before study participation, signed assent forms from all participating subjects ages 7–14 years and signed parental permission forms from their respective parents, and signed consent forms were obtained for all adult study participants. There were no subject withdrawals.

## Author Contributions

CB and GB conceived of this project and defined the prototype *Empower Stars!* video game content. SB, MB, RL, DL, ES, AS, and RS created and coded the story line, characters, exercise mini-game challenges, and music content of the prototype *Empower Stars!* video game, with ongoing consultation from GB and CB. CB and CM designed the Usability Assessment study and survey questionnaires, and GB consulted. CB was responsible for all Institutional Review Board-related documents and processes, wrote the initial manuscript draft, and conducted the Usability Assessment study, with assistance from AB and IP. CB and CM analyzed the usability assessment data. AB and IP contributed to pediatric chronic disease literature review, and all listed coauthors contributed to editing and gave final approval of the manuscript.

## Conflict of Interest Statement

CB and GB are co-inventors on two issued US patents 9,569,562 and 9,747,423 “Disease Therapy Game Technology.” These patents are related to the exercise–empowerment video game technology and are owned by the University of Utah. The remaining coauthors declare that the research was conducted in the absence of any commercial or financial relationships that could be construed as a potential conflict of interest.

## Abbreviations

QOL, quality of life; The PE Game, the Patient Empowerment Exercise Game; eHealth, electronic health; mHealth, mobile health; ADHD, attention deficit hyperactivity disorder.
